# Web-Based Digital Health Interventions for Weight Loss and Lifestyle Habit Changes in Overweight and Obese Adults: Systematic Review and Meta-Analysis

**DOI:** 10.2196/jmir.9609

**Published:** 2019-01-08

**Authors:** Alline M Beleigoli, Andre Q Andrade, Alexandre G Cançado, Matheus NL Paulo, Maria De Fátima H Diniz, Antonio L Ribeiro

**Affiliations:** 1 Internal Medicine Department Faculty of Medicine Universidade Federal de Minas Gerais Belo Horizonte Brazil; 2 Department of Medicine University of Adelaide Adelaide Australia; 3 Flinders Digital Health Research Centre College of Nursing and Health Sciences Flinders University Adelaide Australia; 4 Quality Use of Medicines and Pharmacy Research Center School of Pharmacy and Medical Sciences University of South Australia Adelaide Australia; 5 Telehealth Center Hospital das Clinicas Universidade Federal de Minas Gerais Belo Horizonte Brazil

**Keywords:** internet, mobile phone, meta-analysis, obesity, telemedicine

## Abstract

**Background:**

Obesity is a highly prevalent condition with important health implications. Face-to-face interventions to treat obesity demand a large number of human resources and time, generating a great burden to individuals and health system. In this context, the internet is an attractive tool for delivering weight loss programs due to anonymity, 24-hour-accessibility, scalability, and reachability associated with Web-based programs.

**Objective:**

We aimed to investigate the effectiveness of Web-based digital health interventions, excluding hybrid interventions and non-Web-based technologies such as text messaging, short message service, in comparison to nontechnology active or inactive (wait list) interventions on weight loss and lifestyle habit changes in individuals with overweight and obesity.

**Methods:**

We searched PubMed or Medline, SciELO, Lilacs, PsychNet, and Web of Science up to July 2018, as well as references of previous reviews for randomized trials that compared Web-based digital health interventions to offline interventions. Anthropometric changes such as weight, body mass index (BMI), waist, and body fat and lifestyle habit changes in adults with overweight and obesity were the outcomes of interest. Random effects meta-analysis and meta-regression were performed for mean differences (MDs) in weight. We rated the risk of bias for each study and the quality of evidence across studies using the Grades of Recommendation, Assessment, Development, and Evaluation approach.

**Results:**

Among the 4071 articles retrieved, 11 were included. Weight (MD −0.77 kg, 95% CI −2.16 to 0.62; 1497 participants; moderate certainty evidence) and BMI (MD −0.12 kg/m2; 95% CI −0.64 to 0.41; 1244 participants; moderate certainty evidence) changes were not different between Web-based and offline interventions. Compared to offline interventions, digital interventions led to a greater short-term (<6 months follow-up) weight loss (MD −2.13 kg, 95% CI −2.71 to −1.55; 393 participants; high certainty evidence), but not in the long-term (MD −0.17 kg, 95% CI −2.10 to 1.76; 1104 participants; moderate certainty evidence). Meta-analysis was not possible for lifestyle habit changes. High risk of attrition bias was identified in 5 studies. For weight and BMI outcomes, the certainty of evidence was moderate mainly due to high heterogeneity, which was mainly attributable to control group differences across studies (*R*^2^=79%).

**Conclusions:**

Web-based digital interventions led to greater short-term but not long-term weight loss than offline interventions in overweight and obese adults. Heterogeneity was high across studies, and high attrition rates suggested that engagement is a major issue in Web-based interventions.

## Introduction

Facing the global obesity epidemic is a major public health challenge [1]. The prevalence of obesity has nearly doubled over the last 30 years [1]. Obesity is associated with an increased risk for type 2 diabetes, hypertension, dyslipidemia, cardiovascular diseases, musculoskeletal disorders, psychological stress, and certain types of cancer. All these morbidities significantly increase mortality and reduce quality of life [2].

Obesity treatment involves a systemic approach with both individual and environmental strategies [3]. The individual interventions are usually delivered face-to-face, which generate high demands for individuals, due to their prolonged course, and a great burden to the health care system due to the high prevalence of obesity [4]. Despite such efforts, the effectiveness of obesity interventions on weight loss is only modest, particularly in the long-term [5].

In this context, Web-based digital technology can be a particularly interesting tool for the treatment of overweight and obesity due to its capacity for reaching a large number of people even in remote areas on a 24-hour per 7-day regimen. Delivering weight loss interventions on the Web allows targeting a larger number of people compared to face-to-face interventions and might be less time consuming and more cost-effective for professionals and patients [6]. Previous reviews have shown a modest superiority of digital interventions in comparison to offline interventions with regards to weight loss [7,8]. However, as these reviews included studies that investigated hybrid interventions both in the intervention (eg, Web-based plus short message service text messages) and control groups (ie, face-to-face plus technology-based interventions), the effect of interventions that use only Web-based delivery is not known.

Our aim was to conduct a systematic review and meta-analysis of randomized controlled trials to investigate the effect of Web-based digital interventions in comparison to real-world interventions on anthropometric measures and changes in dietary and physical activity habits in individuals with overweight and obesity.

## Methods

### Systematic Review

For the purpose of this review, PubMed or Medline, SciELO, Lilacs, PsychNet, and Web of Science electronic databases were searched up to July 1, 2018. No language restrictions were applied. We searched both for indexed terms and terms in titles or abstracts that corresponded to the following search pattern in PubMed or Medline: (overweight OR obes*) AND (web OR technology OR internet OR computers OR “social media” OR online).

Studies were eligible if they reported data on randomized controlled trials, which recruited adults (≥18 years) with overweight and obesity (body mass index [BMI] ≥25 kg/m^2^) into a Web-based digital intervention (accessed by browser or Web-based application, regardless of device) versus offline or in-person (face-to-face) interventions. Studies that did not apply any active interventions (wait list) in the control group were also included. Exclusion criteria comprised studies in which overweight and obesity were not a primary selection criterion or those in which the predefined outcomes were not reported. Additionally, studies that included children, adolescents, or pregnant women were excluded. Trials of hybrid interventions (Web-based digital interventions plus face-to-face interventions or other technology-based interventions, such as mobile short message service text messages or digital interventions plus offline interventions) and those that included digital interventions in the control group were also excluded. Moreover, studies evaluating the prevention of weight regain after a previous intervention and those that did not report the predefined outcomes of interest were not included. Multiple reports from the same study were considered as a single one. We considered changes in anthropometric measures and in dietary and physical activity habits as the outcomes of interest.

Two reviewers (AGC and MNLP) independently carried out the selection of the studies according to the predefined eligibility criteria. Any disagreement between them was evaluated by 2 other authors (AMB and AQA). AMB and AQA independently extracted data from reports based on a predefined data extraction form. Any disagreement between them was evaluated by either MdFHD or ALR. When some information was not clear in the report, authors were contacted by email. Hand search was performed in the references of previously published reviews.

### Quality of the Evidence

We used the Grades of Recommendation, Assessment, Development and Evaluation (GRADE) methodology [9] to assess the quality of evidence retrieved by the systematic review. This consists of evaluating the risk of selection (randomization and allocation), performance (blinding of participants and personnel), detection (blinding of outcome assessment), attrition (incomplete outcome data), and reporting (selective reporting) bias of individual studies. In addition, the GRADE methodology suggests assessment of indirectness, inconsistency, imprecision and publication bias of the evidence overall in order to grade the level of the evidence retrieved.

### Meta-Analysis

We used a random effects model to calculate summary mean differences (MDs) and 95% CIs for 1 unit change in weight (kg), BMI (kg/m^2^), waist (cm), or body fat (%). In cases where both “per protocol” and intention-to-treat results were provided, the latter were used to calculate summary MDs. For dietary and physical activity habits, we found a great diversity in the instruments used to measure changes among the groups. This finding precluded meta-analysis, and we performed only qualitative analysis of these outcomes. We used the Cochrane Review Manager software for these analyses [10].

A random effects meta-regression model was used to determine whether the type of control group (with and without active intervention) was a source of heterogeneity among studies. We performed sensitivity analyses according to the length of follow-up (<6 and ≥6 months) and the type of control intervention (presence or not of a nondigital intervention in the control group). We used *Comprehensive Meta-Analysis* software version 3 for this analysis [11].

## Results

### Systematic Review

**Figure 1:**
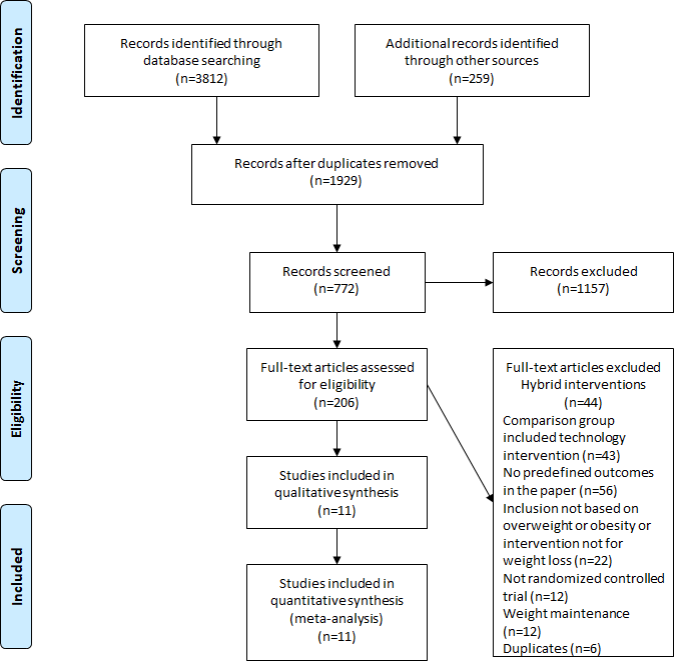
Preferred Reporting Items for Systematic Reviews and Meta-analysis flowchart.

The search strategy resulted in 4071 articles. After exclusions, as shown in Figure 1, a total of 11 studies [12-22] that analyzed data from 1525 participants were retrieved. Female sex was predominant in most of the studies. The age of the participants varied from 18 to 65 years. Most of the studies excluded participants with comorbidities and pregnancy as well as participants who were engaged in other weight loss programs. Unhealthy lifestyle habits were not an inclusion criterion in any of the retrieved studies. Recruitment settings varied among the studies and included community populations, physician-referred patients, company employees, and university students or staff. Other characteristics of the studies retrieved are depicted in Multimedia Appendix 1.

Interventions were predominantly delivered via internet browsers, except 2 that were delivered by a smartphone app [12,16]. Diverse behavioral strategies, such as goal setting, self-monitoring and management, social support, modeling, and feedback were applied in the studies. The control groups received either no intervention (wait list) or usual face-to-face interventions (Table 1).

### Quality of the Evidence

**Figure 2:**
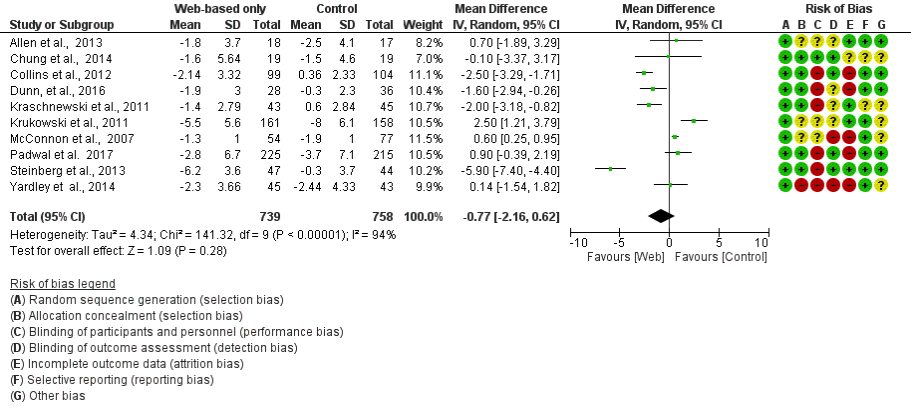
Meta-analysis results for mean weight change (kg) in Web-based-only versus offline interventions. df: degrees of freedom; IV: interval variable; random: random effects model.

**Figure 3:**
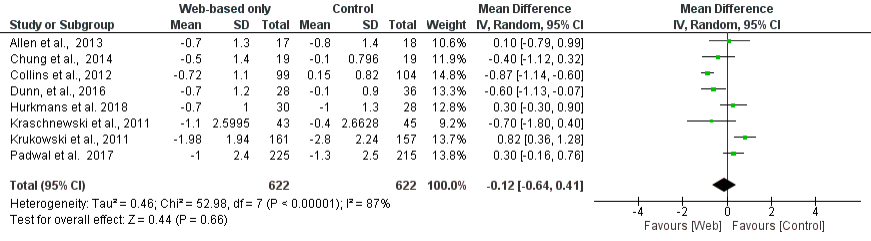
Meta-analysis results for mean body mass index change (kg/m2) in Web-based-only versus offline interventions. df: degrees of freedom; IV: interval variable; random: random effects model.

With regard to the risk of bias of individual studies (Figures 2 and 3), all of the studies reported a sequence generation randomization process. Allocation was not concealed in 3 studies [12,18,20]. As expected in this type of intervention, blinding of participants and personnel was not feasible, whereas blinding of the assessor was not reported in 5 [15,17,19-22]. Moreover, high follow-up attrition rates were a common finding. Moreover, 7 of the 11 retrieved studies showed ≥20% losses to follow-up, and unbalanced losses (intervention>control group) were present in 5 of the 11 studies [12,14,16,18,19].

The quality of the evidence retrieved by the GRADE methodology was considered moderate for the primary outcomes of this review (weight and BMI change), as shown in the summary of findings table (Multimedia Appendix 2). Although indirectness, imprecision, and publication bias were not major issues in this body of evidence, heterogeneity (I^2^=94%; *P*<.001, for weight loss as the outcome) was high and explained mainly (*R*^2^=0.79) by differences in the type of control group as shown by meta-regression analysis.

### Anthropometric Measures

**Figure 4:**
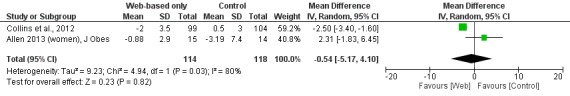
Meta-analysis results for mean waist change (cm) in Web-based-only versus offline interventions for studies with a specific intervention in the control group. df: degrees of freedom; IV: interval variable; random: random effects model.

Absolute weight and BMI changes were reported in 10 [12-15,17-22] and 9 [12-20] studies, respectively. Overall, changes in weight (MD −0.77 kg; 95% CI −2.16 to 0.62; Figure 2) and MDs in BMI (MD −0.12 kg/m^2^; 95% CI −0.64 to 0.41; Figure 3) were not significantly different between the digital interventions and the offline interventions. Only 2 studies reported results on waist circumference [12,14]. There was no difference between the intervention and control groups for this outcome (−0.54 cm; 95% CI −5.17 to 4.10), as shown in Figure 4. Only 1 study reported changes in percent body fat and did not find a significant difference between the intervention and control groups (−1.40%; 95% CI −2.93 to 0.13) [13].

### Lifestyle Habits and Other Outcomes

Among the 11 studies, 8 reported outcomes on dietary or physical activity habit changes [12,14-17,19-21]. However, the instruments used to measure qualitative and quantitative dietary and physical activity characteristics were very different across the studies. This precluded us to perform a quantitative review of these outcomes. Most of the studies reported that there was no significant difference between the intervention and control groups, except for dietary habits in 3 of the studies (Table 2) [14,16,21]. Moreover, 5 studies [14,16,17,20,22] reported data on substitutive measures of cardiovascular morbidity—blood pressure, glucose metabolism, or cholesterol. None of them found any difference between the intervention and control groups. None of the 11 studies investigated hard endpoints, such as cardiovascular morbidity and mortality.

There was no difference between the groups in terms of quality of life in the 3 studies that assessed it [18-20]. The Web-based intervention was cost-effective in comparison to a 6-month in-person intervention in 1 [18] of the 3 studies that evaluated cost-effectiveness [18-20].

**Table 1:** Details of the intervention and control groups.

Study	Behavioral theory	Behavioral strategy	Technology strategy	Type of control intervention	Behavioral strategy in control group	Tools used in control group
Allen et al 2013 [12]	Social cognitive theory, behavioral self-management, and motivational interviewing counseling	Self-management, mindful empowerment, and feedback	Web-based smartphone app	In person	Coaching and goal setting	N/A^a^
Chung et al 2014 [13]	Not specified	Self-monitoring, knowledge, personalized feedback	Image-based electronic portal and caloric calculator	Paper food diary	Self-monitoring	Paper diary
Collins et al 2012 [14]	Social cognitive theory	Self-efficacy, goal setting, and self-monitoring of weight, body measurements, exercise, and diet; outcome expectations (knowledge-based web components); modeling; and social support	Website and telephone contact	Wait list	N/A	N/A
Dunn et al 2016 [15]	Theory of planned behavior, mindfulness, and small steps to change	Web-based lessons of the Eat Smart, Move More, Weigh Less, an evidence-based, 15-week, adult weight management program	Website	Wait list	Theory of planned behavior, mindfulness, and small steps to change	Eat Smart, Move More, Weigh Less, an evidence-based, 15-week, adult weight management program
Hurkmans et al 2018 [16]	Not specified	Knowledge, self-monitoring, help button	Mobile app	In person	Self-monitoring, action planning, relapse prevention	N/A
Kraschnewski 2011 [17]	Positive deviance framework	Modeling, goal setting, knowledge, personalized feedback, self-monitoring	Videos	Wait list	N/A	N/A
Krukowski et al 2011 [18]	Not specified	Knowledge, self-monitoring, stimulus control, problem solving, goal setting, relapse prevention, and assertiveness training	Chat group, pedometers, and website platform	In person	Knowledge, self-monitoring, stimulus control, problem solving, goal setting, relapse prevention, and assertiveness training	Group sessions and printed information on dietary intake and physical activity
McConnon et al 2007 [19]	Not specified	Counseling, personal feedback	Not specified	Primary-care based printed information	Not available	Printed information
Padwal et al 2017 [20]	Not specified	Knowledge, self-management, self-monitoring, goal setting, stress management	Website	In person	Knowledge, self-management, self-monitoring, goal setting, and stress management	N/A
Steinberg et al 2013 [21]	Not specified	Self-monitoring, knowledge and skills (portion control, restaurant eating, structured exercise, problem solving, stimulus control, and relapse prevention)	Cellular-connected “smart” scale for daily weighing, website, and email	Wait list	N/A	N/A
Yardley et al 2014 [22]	Cognitive behavioral theory	Skills, self-regulation, and feedback	Website lessons, challenges, and email	In person	Not available	N/A

^a^N/A: not applicable.

**Table 2:** Differences of lifestyle habits between the intervention and control groups.

Study	Dietary caloric intake	Dietary quality	Physical activity
Allen et al 2013 [12]	No difference	N/A^a^	No difference
Chung et al [13]	N/A	N/A	N/A
Collins et al 2012 [14]	Lower in the intervention group	No difference	No difference
Dunn et al 2016 [15]	No difference	No difference	No difference
Hurkmans et al 2018 [16]	Lower in control group	No difference	No difference
Kraschnewski et al, 2011 [17]	No difference	No difference	No difference
Krukowski et al [18]	N/A	No difference	No difference
McConnon et al [19]	No difference	No difference	No difference
Padwal et al 2017 [20]	N/A	N/A	N/A
Steinberg et al 2013 [21]	Lower in the intervention group	No difference	No difference
Yardley et al 2014 [22]	N/A	N/A	N/A

^a^N/A: not available.

### Sensitivity Analyses

**Figure 5:**
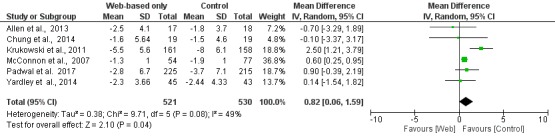
Meta-analysis results for mean weight change (kg) in Web-based-only versus active nontechnology interventions in the control group. df: degrees of freedom; IV: interval variable; random: random effects model.

**Figure 6:**
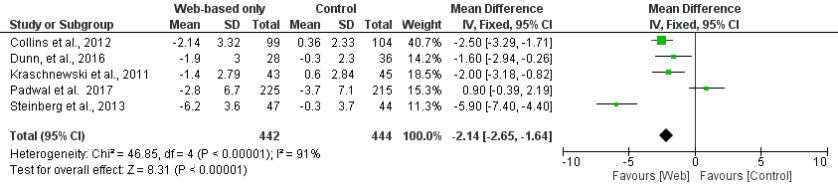
Meta-analysis results for mean weight change (kg) in Web-based-only versus nonactive interventions (wait list) in the control group. df: degrees of freedom; IV: interval variable; random: random effects model.

In the subgroup of studies in which there was an active intervention in the control group, there was a significant difference between Web-based interventions and nontechnology interventions regarding weight loss (MD 0.82 kg; 95% CI 0.06 to 1.59; Figure 5). When the analysis was restricted to the subgroup of studies that did not have any intervention in the control group, the Web-based intervention was superior to control (MD −2.14 kg; 95% CI −2.65 to −1.64; Figure 6).

**Figure 7:**
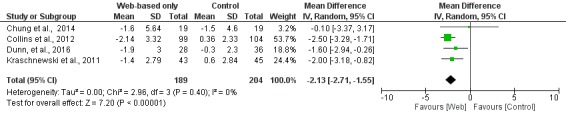
Meta-analysis results for mean weight change (kg) in Web-based-only versus offline interventions for studies with <6 months follow-up duration. df: degrees of freedom; IV: interval variable; random: random effects model.

**Figure 8:**
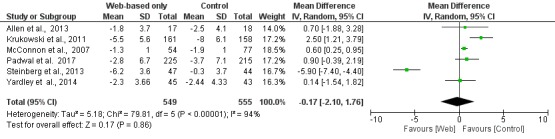
Meta-analysis results for mean weight change (kg) in Web-based-only versus offline interventions for studies with ≥6 months follow-up duration. df: degrees of freedom; IV: interval variable; random: random effects model.

When studies were analyzed according to the length of follow-up, there was greater weight loss (MD −2.13 kg; 95% CI −2.71 to −1.55) in the Web-based intervention group than in the offline intervention group in the subgroup of studies with <6 months of follow-up, whereas there was no difference between the intervention and control groups in the subgroup of studies with ≥6 months of follow-up (MD −0.17 kg; 95% CI −2.10 to 1.76), as shown in Figures 7 and 8, respectively.

## Discussion

### Principal Findings

In this meta-analysis, we found that the use of Web-based digital interventions exclusively was not superior to the use of offline interventions in terms of weight or BMI loss in individuals with overweight and obesity except in the short-term. These findings were based on moderate-quality evidence. Changes in dietary and physical habits of overweight and obese individuals were not different between these 2 types of intervention either.

The findings of superiority of the intervention in comparison to the control for short-term but not long-term weight loss suggest that long-term use and adherence to digital interventions are important issues to consider when planning this kind of intervention. Moreover, the superiority of digital intervention in the subgroup of studies that had no specific intervention in the comparison group suggests that this tool might be more valuable to induce weight loss in patients who do not have access to any kind of in-person intervention.

Intervention-induced weight loss was of small clinical significance. This happened even in studies with a short-term follow-up. Low engagement to the interventions delivered by the Web-based tools might explain these modest results and might be a proxy for the low motivation of participants [23]. These modest results also highlight the need to investigate the components and tools of Web-based platforms that lead to the maintenance of users’ motivation, interest, and participation, which play a key role in enhancing adherence to healthy behaviors.

A great diversity of behavioral techniques was found in the intervention groups across studies. Behavioral strategies with multiple components comprised most of the intervention strategies. This makes it difficult to infer which components are more effective in promoting weight loss and change of health habits and precluded us from identifying whether the results were due to differences in the nature of the interface (Web-based vs face-to-face) or in the behavioral strategy. Additionally, the principles of the interventions applied in the control group were not similar to those applied in the intervention group within each study.

High risk of attrition bias was identified in 5 of the 11 studies. Although most of them followed up participants in the short-term (less than 6 months), loss of ≥20% of participants over the follow-up period was common both in the intervention and control groups. This suggests that Web-based interventions probably do not overcome the low adherence to treatments, which is commonly reported in obesity studies. Another issue of concern regarding the quality of the studies was the scarcity of data on hazardous outcomes related to the weight loss. Since appetite disorders as well as muscle and bone mass reduction may be consequences of weight loss, it was desirable that the studies had included these issues in the results. Differences in the type of control group (with and without intervention) explained a major part of the high heterogeneity found in the meta-analysis.

The thorough revision, which included 5 databases with no language restriction, is a major strength of this study. On the other hand, the high heterogeneity and high risk of attrition bias make recommendations of using Web-based interventions for individuals with overweight and obesity based on their effectiveness on weight loss of moderate certainty.

### Conclusion

There is moderated certainty in our findings that Web-based digital health interventions are more effective than nontechnology interventions in promoting short-term but not long-term weight loss. Moreover, Web-based interventions do not seem superior to nontechnology ones in terms of changes in dietary and physical activities. The high dropout rates in the retrieved studies contributed to a lowered quality of evidence and suggest that designing interventions that maintain participants’ engagement and motivation over time might be fundamental to the success of digital interventions.

## Appendix

Multimedia Appendix 1 Individual study characteristics.

Multimedia Appendix 2 Summary of findings table according to the GRADE methodology.

## Abbreviations

BMI: body mass index
GRADE: Grades of Recommendation, Assessment, Development and Evaluation
MD: mean difference
