# Supplementary material for: Web-Based Digital Health Interventions for Weight Loss and Lifestyle Habit Changes in Overweight and Obese Adults: Systematic Review and Meta-Analysis
Source: J Med Internet Res. 2019 Jan 8;21(1):e298. doi: 10.2196/jmir.9609 (PMC6330028; doi:10.2196/jmir.9609)
Supplement: Multimedia Appendix 1 [file jmir_v21i1e298_app1.pdf]

Multimedia Appendix 1. Individual study characteristics.

Study	BMI range (kg/m²)	Mean age	Female gender (%)	Number of participants in the intervention/control groups	Primary outcome (s)	Secondary outcome (s)	Follow-up (months)	Drop-out rate (%) in the intervention/control group	Setting
Allen et al, 2013 [12]	28-42	45	60	17/18*	Weight		6	41;33	Physician referral
Chung et al 2014 [12, 13]	28-?	37	63	20/20*	Weight	BMI, fat percentage, waist-to-hip ratio, mean arterial pressure	3	5;20	University students and staff
Collins et al 2012 [14]	25-40	42	58	99/104*	BMI	Waist, Blood pressure, heart rate, lipids, glucose, insulin, dietary intake, physical activity	3	25;8	Community
Dunn et al 2016 [15]	≥25	48	86	42/38	Weight, BMI	Confidence in ability of being physically active and eating healthily	4	33;47	State employees
Hurkmans et al 2018 [16]	29-34	45	77	24/21	BMI	Cardio-metabolic risk factors, Dietary patterns, Physical activity	3	25;20	Community
Kraschnewski 2011 [17]	27-40	50	69	50/50	Weight	BMI, Blood Pressure, Quality of Life, total caloric intake	3	10;14	Academic medical center
Krukowski et al, 2011 [18]	25-50	46	93	161/158	Weight		6	0.1;2	Clinical center
McConnon et al 2007 [19]	≥30	46	77	111/110	Weight, BMI		12	51;30	General practice
Padwal et	≥35	40	83	225/215	Weight,	Blood	9	26;32	Clinical

al 2017 [20]					BMI	pressure, Lipids, HbA1c, Quality of life, Readiness to change, Self-efficacy, Mood			center
Steinberg 2013 [21]	25-40	45	80	47/44	Weight	Diet and physical activity behaviors, perceptions of daily self-weighting	6	4;4	University setting
Yardley 2014 [22]	≥30 or ≥28 with hypertension, hypercholesterolemia or diabetes	51	66	45/43	weight	Waist, height, fat mass, blood pressure, fasting lipids, blood glucose and HbA1c	12	30;33	General practice
