# Supplementary material for: Web-Based Digital Health Interventions for Weight Loss and Lifestyle Habit Changes in Overweight and Obese Adults: Systematic Review and Meta-Analysis
Source: J Med Internet Res. 2019 Jan 8;21(1):e298. doi: 10.2196/jmir.9609 (PMC6330028; doi:10.2196/jmir.9609)
Supplement: Multimedia Appendix 2 [file jmir_v21i1e298_app2.pdf]

Multimedia Appendix 2. Summary of findings table according to the GRADE methodology.

Question: Web-based digital health interventions compared to non-technological interventions for weight loss in persons with overweight/obesity

Certainty assessment							Numbe of patients		Effect		Certainty	Importance
Number of studies	Study design	Risk of bias	Inconsistency	Indirectness	Imprecision	Other considerations	Web-based digital health interventions	Nontechnological interventions	Relative (95% CI)	Absolute (95% CI)		
**Weight change**												
10	Randomized trials	Not serious	Serious[b]	Not serious	Not serious	None	739	758	—	MD[a] **0.77 lower** (2.16 lower to 0.62 higher)	⨁⨁⨁◯ MODERATE	IMPORTANT
**BMI change**												
8	Randomized trials	Not serious	Serious[c]	Not serious	Not serious	None	622	622	—	MD **0.12 lower** (0.64 lower to 0.41 higher)	⨁⨁⨁◯ MODERATE	IMPORTANT
**Waist change**												
2	Randomized trials	Serious[d]	Not serious	Not serious	Serious[e]	None	114	118	—	MD **0.54 lower** (5.17 lower to 4.10 higher)	⨁⨁◯◯ LOW	IMPORTANT

**Long-term weight change (≥6 months)**												
6	Randomized trials	Not serious	Serious[f]	Not serious	Not serious	None	549	555	—	MD **0.17 lower** (2.10 lower to 1.76 higher)	⊕⊕⊕◯ MODERATE	CRITICAL
**Short-term weight change (<6 months)**												
4	Randomized trials	Serious[g]	Not serious	Not serious	Not serious	None	189	204	—	MD **2.13 lower** (2.71 lower to 1.55 lower)	⊕⊕⊕◯ MODERATE	IMPORTANT

[a]MD: Mean difference.

[b]High heterogeneity (I^2=94%; *P*<.001), wide variation of point estimates. Heterogeneity was mainly explained by the presence of active interventions in control group and different follow-up times across studies.

[c]High heterogeneity (I^2=87%; *P*<.001), wide variation of point estimates and minimal overlap of confidence intervals. Heterogeneity was mainly explained by the presence of active interventions in control group and different follow-up times across studies.

[d]Large and unbalanced (intervention>control) loss to follow-up rates in the study, which contributed mostly to these results.

[e]Confidence interval of the effect size showed wide variation from large losses to large gains in waist circumference.

[f]High heterogeneity (I^2=94%; *P*<.001), wide variation of point estimates and minimal overlap of confidence intervals

[g]Attrition rates were ≥20% in 3 of the 5 studies that investigated this outcome.
